# Entropy difference-based EEG channel selection technique for automated detection of ADHD

**DOI:** 10.1371/journal.pone.0319487

**Published:** 2025-04-03

**Authors:** Shishir Maheshwari, Kandala N V P S Rajesh, Vivek Kanhangad, U Rajendra Acharya, T Sunil Kumar

**Affiliations:** 1 Department of Electronics and Communication Engineering, Motilal Nehru National Institute of Technology Allahabad, Prayagraj, Uttar Pradesh, India; 2 School of Electronics Engineering, VIT-AP University, Vijayawada, Andhra Pradesh, India; 3 Department of Electrical Engineering, IIT Indore, Indore, Madhya Pradesh, India; 4 School of Mathematics, Physics, and Computing, University of Southern Queensland, Springfield, Queensland, Australia; 5 Department of Electrical Engineering, Mathematics and Science, University of Gävle, Gävle, Sweden; Universiti Tunku Abdul Rahman, MALAYSIA

## Abstract

Attention deficit hyperactivity disorder (ADHD) is one of the common neurodevelopmental disorders in children. This paper presents an automated approach for ADHD detection using the proposed entropy difference (EnD)-based encephalogram (EEG) channel selection approach. In the proposed approach, we selected the most significant EEG channels for the accurate identification of ADHD using an EnD-based channel selection approach. Secondly, a set of features is extracted from the selected channels and fed to a classifier. To verify the effectiveness of the channels selected, we explored three sets of features and classifiers. More specifically, we explored discrete wavelet transform (DWT), empirical mode decomposition (EMD) and symmetrically-weighted local binary pattern (SLBP)-based features. To perform automated classification, we have used k-nearest neighbor (k-NN), Ensemble classifier, and support vectors machine (SVM) classifiers. Our proposed approach yielded the highest accuracy of 99.29% using the public database. In addition, the proposed EnD-based channel selection has consistently provided better classification accuracies than the entropy-based channel selection approach. Also, the developed method has outperformed the existing approaches in automated ADHD detection.

## 1 Introduction

Attention deficit hyperactivity disorder (ADHD) is one of the neurodevelopmental disorders in children. ADHD is characterized by less attention, impulsive nature, and hyperactivity [1]. Generally, it originates in childhood and lasts up to adulthood [2]. The most common symptoms are inattentiveness, repeatedly making the same mistakes, facing difficulty performing tasks and activities, talking too much, or combining inattentive symptoms and hyperactivity symptoms [1]. According to the diagnostic and statistical manual of mental disorders (DSM), it is reported that ADHD affects approximately 3%-5% of school children, and comparatively, boys have more prevalence than girls [3]. In [4], the authors conducted a meta-analysis to estimate the pooled prevalence of ADHD in India. The meta-analysis showed that the overall prevalence of ADHD in India was 7.1% (95% confidence interval: 5.5%-8.8%). The prevalence was higher in boys (8.8%) than in girls (5.1%). Extensive studies state that there is interdependence between ADHD and substance use and abuse, especially in adolescence. Also, lack of attention and impulsivity create an overburden to the parents, family, and society [5]. Therefore, the early recognition of ADHD may help provide proper therapy and, thereby, better living.

Currently, clinicians follow the guidelines of the DSM-fourth edition (DSM-IV) and fifth edition (DSM-V) for ADHD diagnosis [6,7]. This diagnostic process primarily depends on analyzing the subject’s symptoms, discussions with parents, and responses to standardized questionnaires [6,7]. Integrating neural measures into this subjective analysis could provide deeper insights into the condition. For instance, the authors in [8] proposed a multivariate approach to detect ADHD in children by combining parent/teacher-reported executive function (EF) assessments with neural measures of cortical thickness in brain regions associated with EF, using magnetic resonance imaging (MRI). By fusing these data sources and applying machine learning algorithms, they achieved an accuracy of 94.4%. This demonstrates the necessity of quantitative methods that can effectively differentiate ADHD from non-ADHD subjects. Given that ADHD is linked to impairments in inhibitory control and cognitive function, neurophysiological tests hold significant potential for accurate diagnosis [9]. In this context, several studies have utilized neuroimaging techniques, such as MRI [10], as well as physiological signals, including electroencephalogram (EEG) and electrocardiogram (ECG) [7]. Among these methods, EEG stands out as a relatively inexpensive, non-invasive, and efficient tool for studying cognitive changes in the brain [11]. Furthermore, advancements in portable EEG devices have made this method more accessible, enhancing its utility in clinical and research settings.

In the past few decades, several researchers have focused on ADHD detection. As a result, several approaches have been proposed in the literature [12–17]. Lubar et al. explored EEG for ADHD detection [12] in 1991. The studies show that the theta frequency band power to beta frequency band power is higher in ADHD than in healthy controls. In [6], the authors have computed auto-regressive coefficients-based features and are fed to k-nearest neighbor (k-NN) classifier for ADHD detection. They have selected the most discriminated EEG channels based on the accuracy and area under the curve (AUC) values. The proposed method achieved 90% accuracy and 0.98 AUC values. However, only eight subjects of EEG data are used for training and testing, which needs to be increased for more generalization. In [15], nonlinear features, namely, Lyapunov exponent, Higuchi fractal dimension, Katz fractal dimension, and Sevcik fractal dimension, are computed from EEG channels as features, and a multi-layer perception (MLP) is used for classification. This work also proposed an EEG channel selection based on the region of electrode lobes. It stated that for given features and classifier, the frontal region of EEG yields better performance of 96.7% accuracy. The authors’ approach to randomly selecting training and testing groups without proper validation methods may not adequately evaluate the model’s performance. This may lead to data leakage also [18–21].

The approach proposed in [16] is also based on the nonlinear features: fractal dimension (FD), approximate entropy, and Lyapunov exponent and MLP classifier. Here, a minimum Redundancy Maximum Relevance (mRMR) scheme is used for obtaining the most significant features from the whole feature set, and the approach obtained 93.65% accuracy. The major limitation of this work is that the subjects used in this study have not undergone any cognitive assessment to say about any cognitive differences between the two classes of subjects. In recent work, [17], two different decomposition methods, namely wavelet decomposition and empirical mode decomposition, are used as an initial feature extraction step. Later, autoregressive model coefficients, relative wavelet energy, and nonlinear features are extracted, followed by a feature selection method. The sequential forward selection method is employed to reduce the features. Finally, the features are subjected to a k-NN classifier and obtained 97.8% accuracy. This scheme is evaluated on 123 subjects of ADHD and control groups of children. In [22], three multivariate decomposition techniques: multivariate empirical mode decomposition (MEMD), multivariate empirical wavelet transform (MEWT), and multivariate variational mode decomposition (MVMD), are used for ADHD detection. After individually decomposing the EEG signals into subbands using the three methods mentioned above, they computed their instantaneous amplitudes and frequencies. A total of 150 features were extracted, and later using feature selection methods, this feature set was reduced to 15. Using the leave-one-subject-out validation scheme, the authors reported 92 % accuracy when features extracted from MVMD were subjected to the artificial neural network (ANN). The authors mentioned that the EEG channel selection-based scheme could be a potential feature extraction scheme they plan in the future. In [23], the authors proposed a new algorithm called variational mode decomposition and Hilbert transform-based EEG rhythm separation (VHERS) that combines variational mode decomposition (VMD) and Hilbert transform (HT) for separating the EEG signals into different rhythms. The VMD algorithm decomposes the EEG signals into a set of intrinsic mode functions (IMFs), which are then transformed using the HT to extract each rhythm’s amplitude and phase information. The authors use this information to extract features that capture the differences between ADHD and healthy control subjects. The authors evaluated their approach using a dataset of EEG signals collected from 26 ADHD patients and 26 healthy control subjects. They achieved an accuracy of 94.23% in distinguishing between the two groups using a support vector machine (SVM) classifier. A novel eight-pointed star pattern learning network (EPSPatNet86)-based ADHD detection is proposed in [24]. The authors combined convolutional and recurrent neural networks to analyze the EEG signals and extract relevant features. The authors evaluated the performance of the EPSPatNet86 for ADHD detection. They achieved an accuracy of 93.17%, in detecting ADHD. A novel ternary motif pattern (TMP)-based ADHD detection model using EEG signals was proposed in [25]. The authors used TMP to extract features from the EEG signals and then fed them into a machine-learning algorithm for classification. Directed phase transfer entropy (dPTE)-based effective connectivity matrices (ECM) are computed from the EEG signals for identifying children with ADHD [26]. An effective connectivity vector (ECV) is created as a feature vector from ECM computed from different frequency bands of the EEG signals. Later, this feature vector is fed to MLP for classification. The study suggests that analyzing dPTE values from EEG signals may provide a reliable approach to identifying children with ADHD. The authors in [27] decomposed the preprocessed EEG signals into four frequency bands ( theta, alpha, beta, and gamma). Later, these bands are converted to an RGB image. Finally, the images are processed using a convolutional neural network (CNN) to detect ADHD from EEG. The authors in [28] proposed an approach for diagnosing ADHD using nonlinear EEG signal analysis. They computed nonlinear features, including fractal dimension, Hurst exponent, and correlation dimension. The study hypothesizes that nonlinear EEG signal analysis can provide more sensitive and specific measures for diagnosing ADHD than traditional linear measures. The authors in [29] proposed a nonlinear causal relationship estimation by an artificial neural network (nCREANN) method to distinguish between ADHD and healthy children. The nCREANN method estimates the linear and nonlinear patterns of the EEG signal for discriminating the ADHD and healthy subjects.

Channel selection is a process of selecting the most relevant EEG channels that are suitable for an application. Reducing the channels required for automated diagnosis not only reduces the complexity but also helps improve the approach’s performance. Also, this aids in developing portable EEG acquisition systems that may be more convenient for patients [30,31]. Despite the advantages, most of the existing approaches in ADHD detection have not explored channel selection. The approach in [6] has selected the effective and discriminative channels based on the accuracy obtained in ADHD detection. The approach in [15] has investigated the performance channels from different lobe regions for ADHD detection. The authors in [32] developed a model that combines feature selection, decision tree, and logical rule extraction techniques to extract relevant features from the EEG signals and generate an interpretable model for ADHD detection. The authors also analyzed the interpretable model generated by their approach to identify the most relevant EEG features for ADHD detection. They found that the interpretability and explainability of the frontal region are highest compared with the other brain regions. In spite of the success of the above-mentioned approaches, they have the following limitations.

- Recent studies on ADHD detection using EEG are developed based on the different deep learning models. Although the results are promising, these algorithms add complexity to the models for real-time implementation [25,27].
- Despite many advantages of EEG channel selection, including reduced computational complexity and reduced setup time [33], very few approaches [6,15,32] have explored channel selection in ADHD detection. These approaches select EEG channels empirically based on performance metrics, which might not necessarily identify the most informative channels. A systematic approach to channel selection is essential for enhancing accuracy and interpretability.

The main contributions of this work are as follows:

- **Novel Channel Selection Approach:** Introduced a novel channel selection method based on the entropy difference (EnD) value, which systematically identifies the most informative EEG channels for ADHD detection.
- **Automated ADHD Detection Framework:** Designed and implemented a fully automated approach for ADHD detection that leverages the significant channels selected using the EnD-based channel selection method. This framework minimizes computational complexity while maintaining high detection accuracy.
- **Comprehensive Performance Analysis:** Evaluated the effectiveness of the proposed ADHD detection framework using three distinct feature sets across multiple machine learning algorithms. The results demonstrate that the proposed method significantly outperforms existing approaches in terms of accuracy and efficiency for ADHD detection.

The rest of the paper is organized as follows: Sect 2 explains the proposed EnD-based ADHD detection approach. Sect 3 presents results obtained, and finally, conclusions are drawn in Sect 4.

## 2 Methodology

This section briefly describes the proposed EnD-channel selection-based ADHD approach.

**Fig 1:**
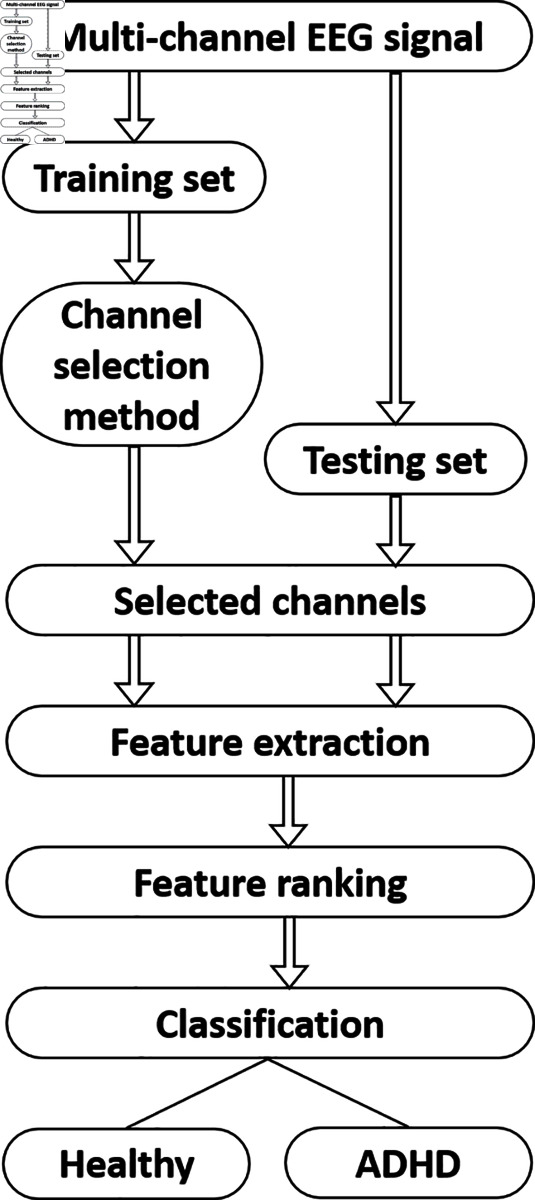
Block diagram of the proposed methodology for the automated detection of ADHD detection from EEG signals.

**Fig 2:**
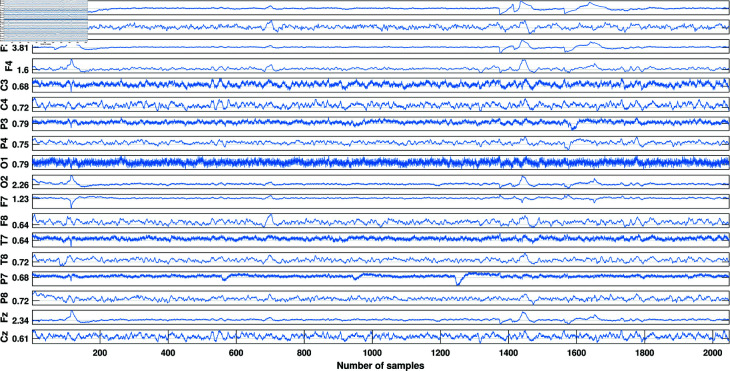
Sample plot of multi-channel EEG segment.

The block diagram of the proposed ADHD detection approach using EnD-based channel selection is shown in Fig 1. Fig 2 shows the sample multi-channel EEG signal. EEG signals are typically high-dimensional, with data collected from multiple electrodes. Firstly, the multi-channel EEG data is divided into train and test sets. Then, the significant channels are selected from the train data based on EnD value, and three sets of features are extracted from each channel. Later, the same set of selected channels is employed in the test set. The features corresponding to individual channels are concatenated to get a final feature vector. After that, the most discriminative features are selected from the extracted feature vector. The rationale behind channel selection is that it can reduce the dimensionality and thereby reduce the overfitting. Besides, not all channels contribute equally to the detection of a specific condition like ADHD. Finally, to test the performance of the proposed approach, the selected features are fed to three different classifiers to detect ADHD.

Furthermore, we have also used entropy-based channel selection to test the effectiveness of our proposed feature selection approach. This method also employed a similar approach as proposed for EnD-based channel selection. The proposed approach is further detailed below.

Entropy-based methods, such as EnD-based channel selection, are particularly useful because entropy quantifies the randomness or complexity of EEG signals, often correlating with neural activity associated with different neurological conditions. By selecting channels based on their entropy values, we ensure that the analysis focuses on the most informative regions of the brain while ignoring irrelevant or redundant data.

### 2.1 Dataset

The dataset employed to assess the performance of the proposed approach is obtained from the IEEE dataport [34,35]. The study comprised 121 children (boys and girls, ages 7-12), with 61 children diagnosed with ADHD and 60 healthy controls. The sample EEG signal is shown in Fig 2. The ADHD diagnosis for the children was confirmed by an experienced psychiatrist using the DSM-IV criteria. They had been taking Ritalin (medicine for treating ADHD) for a maximum of 6 months. On the other hand, none of the children in the control group had any history of psychiatric disorders, epilepsy, or high-risk behaviors. EEG recording was conducted using a 19-channel system, with electrode placements following the 10-20 standard. The recording included 19 channels, specifically Fz, Cz, Pz, C3, T3, C4, T4, Fp1, Fp2, F3, F4, F7, F8, P3, P4, T5, T6, O1, and O2, at a sampling frequency of 128 Hz. The A1 and A2 electrodes on the earlobes served as the reference electrodes for the recording with a sampling frequency of 128 Hz.

The EEG recording protocol utilized for data recording is visual attention tasks. In the task, children were presented with pictures featuring cartoon characters and asked to count the number of characters in each image. The number of characters varied randomly between 5 and 16, and the pictures were sized appropriately to allow for easy counting. To maintain a continuous stimulus during EEG recording, each image was displayed without interruption immediately after the child’s response. Consequently, the duration of the EEG recording for this cognitive visual task depended on the child’s response speed.

### 2.2 Entropy difference (EnD) based method for channel selection

Entropy-based channel selection: Before introducing EnD-based channel selection, we briefly reviewed the entropy-based channel selection. As the name suggests, the channel selection here is based on entropy value. Entropy is a measure of randomness or uncertainty of an EEG channel [36]. The entropy of channel *C* is given as [36]:


$$\begin{array}{ccc} H(C)=-\overset{n}{\underset{i=1}{\sum }}p(x_{i})log_{2}p(x_{i}) & & \end{array}$$ (1)

*p*(*x_i_*) is the probability mass function of the EEG channel with *n* samples. After computing entropies of all the EEG channels, the first *N* channels with the highest entropy are selected for ADHD detection.

EnD-based channel selection: To select the most effective channels for ADHD detection, we develop EnD-based channel selection. The entropy difference of channel *C* is computed as follows:


$$\begin{array}{ccc} EnD(C)=abs(H_{ADHD}(C)-H_{HC}(C)) & & \end{array}$$ (2)

After computing EnD, we selected the top *N* channels based on the maximum entropy difference. Here, *H_ADHD_*(*C*) represents the entropy of EEG channel *C* of ADHD in training data, and *H_HC_*(*C*) represents the entropy of EEG channel *C* of healthy control in training data. The significance of the EnD is that it represents the change in information due to ADHD. More specifically, high EnD for a channel represents a significant change in information due to ADHD, and low EnD represents less change in information.

### 2.3 Feature Extraction

This section presents brief details about the extracted features from the EEG segments.

#### Discrete wavelet transform-based features

The discrete wavelet transform (DWT) is a mathematical technique that provides a time-frequency representation of signals [37]. In the wavelet transform, a signal is decomposed into a series of wavelet coefficients representing different scales and positions. The wavelet coefficients at each scale and position provide information about the signal’s frequency content at a particular time. It allows the wavelet transform to capture the signal’s time and frequency-domain features simultaneously.

The db4 wavelet has become a popular choice for wavelet transforms due to its good balance between temporal and frequency resolution and its ability to handle signals effectively with abrupt changes [38,39]. The EEG segments are decomposed into three decomposition levels using the db4 mother wavelet in this proposed approach. Further from each sub-component, four statistical features, such as Shannon entropy, mean, variance, and energy, are extracted.

#### EMD-based features

EMD is a time-frequency technique that decomposes a non-stationary signal into a finite number of modes known as IMFs [40]. Each IMF consists of a narrow band frequency and is extracted without any prior knowledge of the underlying frequencies. Unlike wavelets, EMD decomposes signals adaptively based on the frequency component of the signal. It also does not employ any basis function as used by wavelets. A signal *S*(*t*) can be decomposed into IMFs as follows [40]:


$$\begin{array}{ccc} S(t)=\overset{N}{\underset{n=1}{\sum }}(IMF{)}_{n}(t)+Res_{N}(t) & & \end{array}$$ (3)

where *S*(*t*) is the multi-component signal. *IMF_n_*(*t*) is the *n^th^* IMF and *Res_N_*(*t*) represents the residue corresponding to *N* intrinsic modes. Each IMF satisfies two conditions: (1) the number of extrema and zero-crossings must be equal or differ by at most one, and (2) the mean value of the envelope defined by the local maxima and minima must be zero. More mathematical details of EMD can be found in [41].

In this work, the EEG segments are decomposed into 3 IMFs using EMD. As mentioned in the above sub-section, the same statistical features are extracted from these IMFs.

#### SLBP-based features

SLBP-based features are time-domain features. It is a local descriptor, which is found to be effective in the classification of EEG signals [42]. In SLBP computation, a binary string is generated for every sample in a signal. The binary string is generated by comparing a sample’s right and left neighborhoods. Thus, a binary string is converted into a decimal number using a symmetrical weighting scheme. By computing SLBP-based features for all the samples in a signal, we can obtain a feature vector that captures the local variations of the signal. SLBP is mathematically computed as follows [42]:


$$\begin{array}{ccc} {SLBP}_{LHS}(x[n]) & =\overset{L-1}{\underset{m=0}{\sum }}f(x[n+m-L]-x[n])2^{L-1-m} & \end{array}$$ (4)

$$\begin{array}{ccc} {SLBP}_{RHS}(x[n]) & =\overset{L-1}{\underset{m=0}{\sum }}f(x[n+m+1]-x[n])2^{L} & \end{array}$$ (5)

$$\begin{array}{ccc} SLBP(x[n]) & ={SLBP}_{LHS}(x[n])+{SLBP}_{RHS}(x[n]) & \end{array}$$ (6)

where *x*[*n*] represents the current sample of the signal, *L* is the neighborhood size considered on either side of the current sample, and threshold function *f* is defined as


$$\begin{array}{ccc} f(x)=\{\begin{array}{ccc} 0, & & x<0\\ 1, & & x\geq 0 \end{array} & & \end{array}$$ (7)

Sample SLBP computation can be found in [43]. From each EEG segment, the SLBP provides 31 features.

### 2.4 Feature selection using Chi-square test

The performance of the computer-aided system significantly depends on the features extracted from the data. Each feature does not contribute equally, and features with low inter-class discriminating capability hamper the system’s performance. The feature selection method provides each feature’s discriminating value, which can be used to select valuable features. In this work, we employed a Chi-square test for feature selection [44]. The Chi-square test evaluates the relationship between each feature and the response variable (i.e., the class label). The test measures the dependence of the feature and the response by comparing the observed frequency distribution of the feature for each class to the expected frequency distribution under the assumption of independence. If the Chi-square statistic exceeds the critical value, the feature is considered to have a significant relationship with the response and is selected as a discriminatory feature [44].

### 2.5 Classifiers

This subsection details the machine learning algorithms used for the automated detection of ADHD using EEG signals.

#### Support Vector Machine (SVM)

SVM is a supervised classifier proposed by Vapnik [45]. SVMs are naturally binary classifiers, and they construct a hyperplane in the higher dimensional feature space to separate the boundaries between feature vectors from both classes. Finding the best hyperplane that gives the highest margin distance between the nearest feature vectors of the two class labels is an optimization problem. The main advantage of this algorithm is that it can also be extended to multiclass classification by following one-against-all or one-against-one techniques. Using a kernel method, nonlinear data can be separated in a higher dimensional space. More details can be found in [46].

#### k-nearest neighbor (k-NN)

k-NN is another supervised learning algorithm like SVM. Unlike SVM, k-NN is instance-based learning. This means that k-NN uses whole data at a time for training instead of learning some parameters, such as SVM. Hence, k-NN is also known as non-parametric learning. k-NN stores the entire dataset in the memory and tests a new sample (feature vector) with k nearest (closest in the distance) training feature vectors in the memory and allocates the class label using a majority voting scheme. The often-used distance metrics are Euclidean distance and cosine similarity. More details can be found in [47].

#### Ensemble learning

Ensemble learning models are suitable for nonlinear data processes [47]. The ensemble learning method trains various low-accuracy models and clubs their predictions to get the final prediction result. It gives better generalization and accurate results and alleviates the problem of finding a unique, accurate model for solving the given problem. Moreover, learning a weak model reduces complexity and increases the speed of the process. More details of ensemble learning models can be found in [47].

## 3 Results

This section presents the details of the experimental results. The experiments were carried out in MATLAB version 2023b environment on the system with Intel i5 processor with 12 GB RAM. The default classifier parameters are used for the experiments.

**Algorithm 1: Algorithm for 70:30 split validation strategy**

1: dataset = load_dataset(path-to-dataset)

2: train_set, test_set = divide_dataset(dataset, split_ratio)

3: **for each ** channel ** in ** train_set **do**

4: channel_rank = rank_entropy_diff(channel)

5: **endfor**

6: data_ranked = rank_data(data, channel_rank) {Here data is train_set or test_set}

7: segmented_data = [ ]

8: **for each ** channel ** in** range(19) **do**

9: segments = segment_channel(data_ranked[channel], segment_length)

10: segmented_data.append(segments)

11: **endfor**

12: selected_channels = select_best_ranked_channels(channel_rank, top_k=3)

13: ch_feat = [ ]

14: **for each ** channel ** in ** selected_channels **do**

15: seg_feat = [ ]

16: **for each ** segment ** in ** segmented_data[channel] **do**

17: seg_feat.append_vertically(extract_features(segment))

18: **endfor**

19: ch_feat.append_horizontally(seg_feat)

20: **endfor**

21: ch_feat_chiranked = chisquare_feature_selection(ch_feat)

22: model_classifier = train_classifier(classifier_type, ch_feat_chiranked)

23: results = evaluate_classifier(model_classifier, test_set)

### 3.1 Experimental results

In this section, we presented the experimental conditions and results of our proposed method in Fig 1. To demonstrate the effectiveness of the EnD-based channel selection approach, we utilized three distinct feature extraction methods: EMD, DWT, and SLBP-based feature extraction approaches.

To identify ADHD from EEG signals, we employed three different supervised classifiers: SVM with RBF kernel, k-NN, and Ensemble Learning (ENS).

To evaluate the performance of our approach, we utilized three validation strategies and are given below:

1. 70:30 split ratio: The dataset was split into a 70% training set and a 30% testing set. The classification model was trained on the training set and evaluated on the testing set.
2. 70:30 random split evaluation: The 70:30 split evaluation was repeated ten times, each time with a different random split. The results from these ten evaluations were averaged to obtain more robust and representative performance measures.
3. Ten-fold cross-validation: The dataset was divided into ten folds, each serving as a testing set once, while the remaining nine folds are used for training. This process was repeated ten times, ensuring each fold acted as the testing set once. The performance metrics obtained from these ten iterations were combined to assess the classification model’s overall effectiveness.

In this work, we have used accuracy, sensitivity and specificity parameters to evaluate the performance of the proposed ADHD approach [27].

#### 70:30 split ratio

This validation strategy is provided in Algorithm 1. In the 70:30 split evaluation setup, firstly, the dataset is divided into training (the first 70% of the data) and testing (the last 30% of the data) subsets. The EEG signals used in this study are collected from multiple channels, capturing information from different brain lobes. Each channel provides distinct brain information. Consequently, we conducted a channel-based analysis for ADHD diagnosis. To accomplish this, our method’s first step is assigning ranks to all 19 channels of each EEG signal using training data. Two approaches were employed: entropy(En)-based channel ranking and entropy difference (EnD)-based channel ranking techniques. The ranks obtained from these two methods, ranging from high (1) to low (19), are presented in Tables 1 and 2.

**Table 1:** Ranking obtained using the entropy-based channel selection approach.

S. No.	Channel	S. No.	Channel	S. No.	Channel	S. No.	Channel
	name		name		name		name
1	Cz	6	O2	11	Pz	16	F7
2	F4	7	P7	12	T8	17	Fp2
3	P8	8	F3	13	F8	18	Fz
4	P4	9	O1	14	P3	19	Fp1
5	C4	10	C3	15	T7		

**Table 2:** Ranking obtained using the EnD-based channel selection approach.

S. No.	Channel	S. No.	Channel	S. No.	Channel	S. No.	Channel
	name		name		name		name
1	Fz	6	P3	11	P4	16	Fp2
2	Pz	7	F3	12	T8	17	C4
3	P7	8	Fp1	13	Cz	18	F4
4	O1	9	C3	14	O2	19	P8
5	T7	10	F7	15	F8		

**Fig 3:**
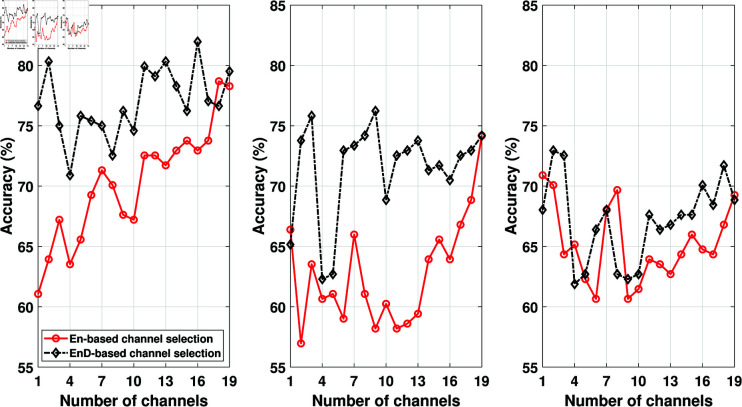
Comparison of ENS (left), k-NN (middle), and SVM (right) classifier accuracy obtained from EMD-based features for EN- and EnD-based channel selection method (before feature selection).

**Fig 4:**
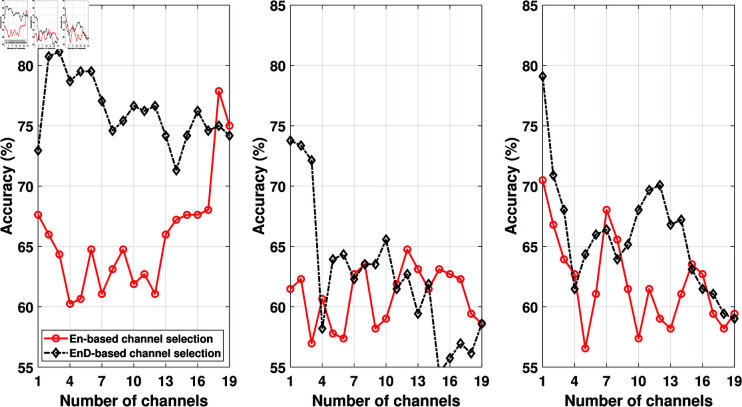
Comparison of ENS (left), k-NN (middle), and SVM (right) classifier accuracy obtained from DWT-based features for En- and EnD-based channel selection method (before feature selection).

**Fig 5:**
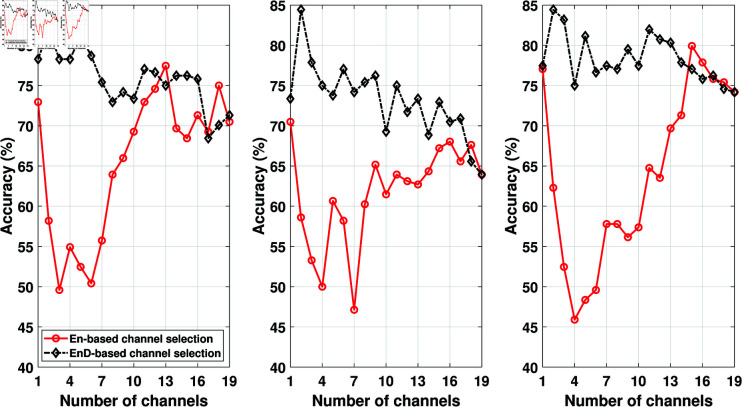
Comparison of ENS (left), k-NN (middle), and SVM (right) classifier accuracy obtained from SLBP-based features for En- and EnD-based channel selection method (before feature selection).

After obtaining the channel ranking from training data, the test data channels have also been arranged based on the channel ranking obtained from training data. After that, we divided each channel into segments of 2048 (16 seconds) samples and obtained the features from each segment. To compare the EnD and entropy-based channel selection approaches, we plotted the classification accuracy against the number of channels used in ADHD detection. The results obtained are depicted in Figs 3, 4, and 5. Our primary focus was to analyze the performance of the EnD and entropy-based channel selection approaches, so no additional feature selection was applied to these results. The figures show that the channel ranking obtained using the EnD-based method consistently outperformed the entropy-based method across all employed feature extraction and classification methods. Notably, we observed that after the first three best-ranked channels, there was no significant improvement in classification accuracy in the case of the EnD-based channel selection scheme. Furthermore, increasing the number of channels would result in longer feature vectors, potentially leading to increased complexity.

We considered the feature vectors derived from the first three best-ranked channels to facilitate further analysis. The lengths of the features computed from the selected channels using the various methods mentioned above are presented in Tables 3, 4, and 5. The decomposition level and number of intrinsic mode functions (IMFs) for wavelet transform and EMD, respectively, are fixed at 3.

**Table 3:** Feature vector length used for EMD-based method.

Number of channels (NCh)	Feature length
1	12
2	24
3	36

**Table 4:** Feature vector length used for Wavelet-based method.

NCh	Feature length
1	16
2	32
3	48

**Table 5:** Feature vector length used for SLBP-based method.

NCh	Feature length
1	31
2	62
3	93

Therefore, we considered the feature vectors from the first three best-ranked channels for further analysis. To further reduce feature vector length, features extracted from the selected channels are given to the Chi-square test-based feature selection approach. These features are then fed into various supervised classifiers, namely SVM (RBF kernel), k-NN, and ENS, to discriminate between ADHD and healthy subjects. The performance measures for this experimental setup are presented in Tables 6, 7, and 8. Tables 6, 7 and 8 represent the feature extraction using the EMD, wavelets, and SLBP-based methods, respectively.

**Table 6:** Results obtained with EMD-based features using 70:30 split ratio strategy.

Classifier	NCh	Acc in %		Sn, Sp in %	
		En	EnD	En	EnD
ENS	1	65.98	80.73	61.05, 69.12	78.94, 81.87
	2	72.95	83.61	58.94, 81.87	85.26, 82.55
	3	72.95	83.61	58.94, 81.87	85.26, 82.55
k-NN	1	66.39	76.64	62.10, 69.12	82.10, 73.15
	2	65.98	76.64	64.21, 67.11	82.10, 73.15
	3	67.21	76.64	54.73, 75.16	82.10, 73.15
SVM	1	71.31	78.27	80.00, 65.77	90.52, 70.46
	2	72.13	81.55	80.00, 67.14	93.68, 73.82
	3	71.72	81.55	73.68, 70.46	92.63, 74.49
En: Entropy-based channel selection					
Acc: Classification accuracy, Sn: Sensitivity, Sp: Specificity					

**Table 7:** Results obtained with DWT-based features using 70:30 split ratio strategy.

Classifier	NCh	Acc in %		Sn, Sp in %	
		En	EnD	En	EnD
ENS	1	69.67	79.50	43.15, 86.57	71.57, 84.56
	2	67.21	88.11	47.36,79.86	84.21, 90.60
	3	64.75	87.29	47.36, 75.83	88.42, 86.57
k-NN	1	65.98	75.00	55.78, 72.48	86.31, 67.78
	2	62.29	84.83	56.84, 65.71	81.05, 87.24
	3	62.29	83.60	50.52, 69.79	81.05, 85.23
SVM	1	77.86	85.24	83.15, 74.49	85.26, 85.23
	2	71.72	88.52	86.31, 62.41	97.89, 82.55
	3	72.13	88.52	74.73, 70.46	93.68, 85.23

**Table 8:** Results obtained with SLBP-based features using 70:30 ratio strategy.

Classifier	NCh	Acc in %		Sn, Sp in %	
		En	EnD	En	EnD
ENS	1	72.95	79.09	65.26, 77.85	67.36, 86.57
	2	66.39	85.24	57.89, 71.81	82.10, 87.24
	3	63.52	84.83	57.89, 67.11	73.68, 91.94
k-NN	1	70.90	73.36	70.52, 71.14	69.47, 75.83
	2	58.60	84.42	58.94, 58.38	83.15, 85.23
	3	58.19	79.09	67.36, 52.34	78.94, 79.19
SVM	1	78.27	79.91	72.63, 81.87	72.63, 84.56
	2	64.34	85.65	62.10, 65.77	80.00, 89.26
	3	59.01	85.65	46.31, 67.11	82.10, 87.91

Overall, comparing EnD and entropy-based channel selection approaches showed that EnD consistently outperformed entropy channel selection in ADHD detection across different feature extraction and classification methods. Limiting the feature vectors to the top three ranked channels also offered a good balance between accuracy and complexity, leading to more efficient and effective ADHD detection.

#### 70:30 random split ratio strategy

In this evaluation, a different approach is taken compared to the previous 70:30 split ratio strategy. The dataset is randomly divided into training (70%) and testing (30%) sets, and this process is repeated ten times. The purpose of this method is to obtain more reliable performance metrics.

The results of these repeated evaluations for EMD-based features, wavelet-based features, and SLBP-based features are presented in Table 9. From this table, it can be observed that the wavelet-based features exhibited the highest accuracy, reaching 95.68% with ENS classifier. The performance of wavelet-based features with the EnD approach exhibited the potential for effective ADHD classification.

**Table 9:** Average performance metrics obtained with 70:30 random split.

Feature	Classifier	NCh	Acc in %		Sn, Sp in %	
			En	EnD	En	EnD
EMD-based	ENS	1	75.93	79.23	68.932, 81.15	85.34, 74.30
		2	84.42	85.38	85.58, 83.45	89.65, 81.94
		3	85.24	85.24	82.88, 87.21	80.18, 89.47
	k-NN	1	67.39	75.76	59.16, 73.71	80.17, 72.22
		2	74.07	81.14	65.87, 81.25	76.57, 84.96
		3	75.00	83.07	73.33, 76.28	80.17, 85.41
	SVM	1	75.00	81.53	61.26, 86.46	87.93, 76.38
		2	85.55	83.92	93.65, 78.4722	80.35, 86.71
		3	84.81	86.30	95.23, 75.6944	95.23, 78.47
DWT-based	ENS	1	79.50	82.08	70.27, 87.21	83.33, 80.95
		2	85.24	92.15	89.18, 81.95	84.82, 97.90
		3	89.75	95.68	85.58, 93.23	91.07, 99.30
	k-NN	1	75.41	76.92	77.19, 73.80	80.17, 74.30
		2	77.17	86.88	77.50, 76.92	81.08, 91.72
		3	81.11	86.88	80.95, 81.25	81.08, 91.72
	SVM	1	79.60	85.84	73.21, 84.61	83.63, 88.07
		2	90.37	87.09	99.20, 82.63	93.80, 81.48
		3	87.40	87.05	97.61, 78.47	97.61, 78.47
SLBP-based	ENS	1	79.66	81.27	71.84, 85.50	72.72, 89.90
		2	81.85	85.60	78.99, 84.49	87.37, 84.41
		3	85.08	87.65	76.47, 93.02	79.79, 93.38
	k-NN	1	77.17	78.73	66.99, 84.78	71.13, 84.67
		2	82.19	88.75	86.36, 77.98	82.45, 94.44
		3	84.78	90.39	79.16, 89.10	91.08, 89.84
	SVM	1	84.23	83.26	77.66, 89.13	79.61, 85.71
		2	84.47	89.16	90.00, 78.89	85.96, 92.06
		3	88.76	91.05	90.83, 87.17	87.27, 94.11

#### 10-fold cross-validation

In addition to the previous evaluation methods, we also conducted *k*-fold cross-validation strategies to assess the effectiveness of our proposed method. In this validation scheme, the dataset was divided into *k* folds, with one-fold as the testing set, while the remaining *k*–1 folds were used for training. This process was repeated *k* times, and the results were aggregated to provide a comprehensive performance measure. In our study, we employed 10-fold cross-validation, dividing the dataset into ten folds. The performance measures obtained using a 10-fold cross-validation strategy are presented in Tables 10, 11, and 12, following the same format as the previous evaluation schemes. From these tables, it is evident that the SLBP-based features achieved the highest accuracy, reaching 99.29% with the k-NN classifier.

**Table 10:** Results obtained with EMD-based features using 10-fold cross-validation strategy.

Classifier	NCh	10-fold Acc in %		10-fold Sn, Sp in %age	
		En	EnD	En	EnD
ENS	1	77.92	81.32	68.96, 84.94	75.36, 86.02
	2	84.74	90.35	79.44, 88.87	86.06, 93.73
	3	90.35	93.87	86.30, 93.53	92.01, 95.33
k-NN	1	73.79	73.58	70.79, 76.15	69.65, 76.71
	2	80.13	85.33	74.91, 84.23	79.92, 89.59
	3	87.04	91.36	85.15, 88.53	88.58, 93.53
SVM	1	77.21	78.70	75.33, 78.68	75.79, 80.99
	2	80.80	86.25	77.82, 83.14	86.52, 86.02
	3	83.13	88.05	83.78, 82.61	89.70, 86.75

**Table 11:** Results obtained with Wavelet-based features using 10-fold cross-validation strategy.

Classifier	NCh	10-fold Acc in %		10-fold Sn, Sp in %	
		En	EnD	En	EnD
ENS	1	89.46	89.26	82.58, 94.08	86.08, 91.75
	2	93.16	96.68	89.03, 96.40	95.19, 97.84
	3	95.58	97.68	92.93, 97.67	96.81, 98.38
k-NN	1	83.63	84.84	81.28, 85.47	80.80, 87.98
	2	93.47	93.77	92.46, 94.27	92.23, 94.99
	3	96.08	96.68	95.44, 96.58	96.12, 97.12
SVM	1	83.32	85.94	83.12, 83.50	86.03, 85.83
	2	90.56	92.07	92.02, 89.43	91.55, 92.49
	3	90.25	92.16	91.78, 89.24	92.43, 91.93

**Table 12:** Results obtained with SLBP-based features using 10-fold cross-validation strategy.

Classifier	NCh	10-fold Acc in %age		10-fold Sn, Sp in %age	
		En	EnD	En	EnD
ENS	1	81.52	85.44	75.10, 86.55	82.19, 87.99
	2	87.45	94.87	83.55, 90.50	92.92, 96.40
	3	91.67	96.48	88.83, 93.90	95.88, 96.95
k-NN	1	83.54	84.43	81.05, 8549	78.34, 89.25
	2	95.38	97.89	94.30, 96.24	96.58, 98.92
	3	99.19	99.29	98.86, 99.64	99.09, 98.92
SVM	1	85.54	86.44	82.64, 87.81	82.39, 89.61
	2	92.06	96.38	88.57, 94.79	94.30, 98.03
	3	95.98	97.79	95.43, 96.40	97.48, 98.02

### 3.2 Discussion

Considering the results obtained from all three validation schemes, it may be noted that the wavelet-based method and SLBP consistently outperformed the EMD-based approach. Interestingly, the SLBP-based method produced accuracy results comparable to the wavelet-based technique while being computationally more efficient. This is because the SLBP method does not require signal decomposition but operates on neighboring samples, and it does not necessitate the computation of additional features as required in the wavelet and EMD methods. Also, it can be inferred that EnD-based channel selection effectively selects the useful channels in ADHD detection.

**Fig 6:**
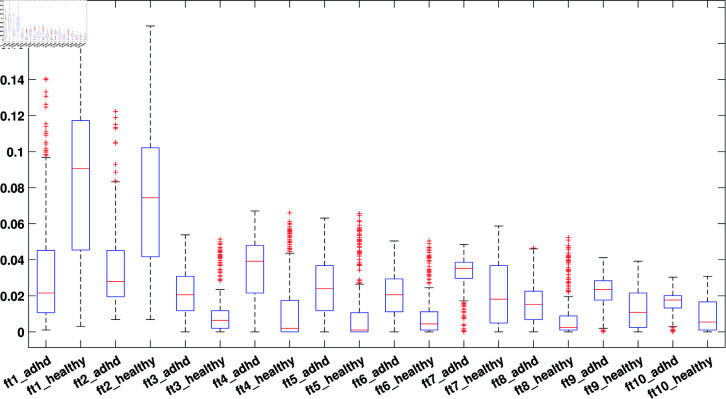
Box plot of first 10 SLBP-based features obtained from first 3 EnD channels of the training set.

**Fig 7:**
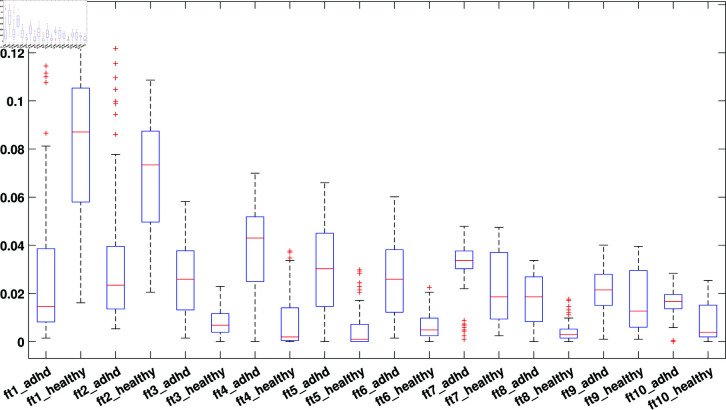
Box plot of first 10 SLBP-based features obtained from first 3 EnD channels of the testing set.

To assess the discriminative ability of features extracted from EnD-based channels, we conducted a statistical analysis (with only SLBP features). Box plots were generated for the first 10 SLBP features, representing the training and testing sets, as depicted in Figs 6 and 7, respectively. These plots demonstrated that the median values of these features are distinct and well separated between the two sets.

The performance comparison of the proposed approach with existing EEG-based ADHD detection methods is shown in Table 13. The table shows that the proposed approach has outperformed the existing approaches in ADHD detection. The work in [25] tried to mimic a deep learning model by using TMP and tunable Q-wavelet transform (TQWT) for extracting the significant features. However, employing a subband decomposition scheme imposed additional complexity on the model. They obtained the highest accuracy using ten-fold cross-validation. In work [24], the EEG segments were decomposed into 85 subband coefficients using TQWT and wavelet packet decomposition (WPD) techniques. Later, 86 handheld feature set is extracted from the subbands. Again, it is a complex model that involves time-frequency approaches and a vast feature generation process. The authors also reported their highest accuracy using a 10-fold cross-validation scheme. The approach in [26] also employed EEG signal segmentation into its individual frequency bands for feature extraction. They also employed ANN and genetic algorithms for ADHD classification. The work in [27] employed deep learning methods for classification purposes. Employing deep neural networks has limitations like increased computational complexity and lack of generalization when training data is limited. The primary advantages, limitations, and future works are discussed below:

#### Advantages

- The proposed method significantly reduces computational load by utilizing features from only a subset of EEG channels instead of all channels. This reduction allows for faster processing, making the approach suitable for real-time applications and enhancing its practicality.
- Even with fewer channels, the method sustains high performance in detecting ADHD. Additionally, the EnD-based approach consistently outperforms the traditional En-based approach, demonstrating its effectiveness.
- The EnD-based channel selection offers an objective and systematic framework for identifying the most informative EEG channels, minimizing subjective biases typically associated with channel selection.
- Notably, the EnD-based method achieves an accuracy of 99.29% in ADHD detection using features derived from just the top three significant channels.

#### Limitations

- The primary limitation of our approach is that the number of channels to be used must be specified manually.
- Although we have mathematically identified the significant channels, it is crucial to establish their medical significance before applying them for clinical purposes.
- Furthermore, the experiments conducted in this study were performed using a single dataset.

#### Future Scope

- The future direction involves incorporating deep learning techniques to enhance the performance of the approach further.
- The EnD-based channel selection technique can be employed for autism, epilepsy, Parkinson’s disease, etc.
- Future efforts could focus on automating the determination of the optimal number of EEG channels required. This can be achieved by developing algorithms that adaptively adjust the channel count based on data characteristics.
- We also plan to detect ADHD using electrocardiogram (ECG) signals using our proposed method [48].
- We also believe that data fusion is another good option to build an effective decision support system [49].
- Also, explainable artificial intelligence (XAI) and uncertainty quantification can be used to evaluate the influence of noise on the developed model [50].

**Table 13:** Performance comparison with existing automated ADHD detection approaches developed.

Paper	Approach	Accuracy (%)	Dataset used
[25]	Tunable-Q wavelet transform-based	95.57	61 ADHD
	features along with kNN classifier		60 HC
[24]	Eight-pointed star pattern	97.19	61 ADHD
	learning network		60 HC
[26]	Directed Phase Transfer	89.2	61 ADHD
	Entropy (dPTE) based approach		60 HC
[27]	RGB Image extracted from	97.47	61 ADHD
	frequency bands are fed to CNN		60 HC
[28]	Non-linear features extracted from	96.05	50 ADHD
	EEG signals are fed to SVM classifier		26 HC
[29]	nCREANN	99	61 ADHD
			60 HC
[51]	Gabor filter-based statistical features	96.05	61 ADHD
			60 HC
-	Proposed approach	88.52 (70:30 split)	61 ADHD
		95.68 (70:30 split performed 10 times)	60 HC
		99.29 (10-fold cross-validation)	

## 4 Conclusion

This study presents an automated method for ADHD detection utilizing an EnD-based channel selection algorithm. The proposed approach achieved an accuracy of 99.29% in identifying ADHD, even when limited to using only the top three most significant channels for classification. This reduction in the number of channels substantially lowers computational complexity, making the method highly efficient. Moreover, the EnD-based channel selection proved to be more effective than traditional entropy-based selection methods in enhancing the performance of automated ADHD detection.

In the future, we aim to extend this approach to identify significant channels for applications such as epilepsy detection and brain-computer interfaces. Moreover, we will focus on leveraging large-scale EEG datasets collected from multiple centers to validate further and expand the utility of the proposed method.
